# Morphology and Chemical Composition of Magnetic Particles Separated from Coal Fly Ash

**DOI:** 10.3390/ma15020528

**Published:** 2022-01-11

**Authors:** Tadeusz Czech

**Affiliations:** Institute of Fluid Flow Machinery, Polish Academy of Sciences, Fiszera 14, 80-231 Gdańsk, Poland; plasdyn@imp.gda.pl

**Keywords:** fly ash, magnetic particles, electrostatic precipitator, coal combustion

## Abstract

Iron and other metal compounds are the materials that often appear in coal seams, because they also appear as a component of former organic matter in coal rocks. Although iron is the dominant element in coal rocks, other metals such as titanium, lead, cobalt, nickel, and copper are also present. In this study, the properties of magnetic particles of a size between 1 and 20 µm of globular structure and iron containing, were separated from coal fly ash, and studied using a scanning electron microscopy, energy disperse spectroscopy, and X-ray diffraction spectroscopy. The investigations were comprised of micrographs of the structure of these particles, their elemental composition, and phase analysis.

## 1. Introduction

Coal is a primary source of energy that is widely used to generate electricity. About 41% of the global electricity is produced by thermal power plants all over the world. Fly ash and bottom ash are produced from coal combustion as a waste product in large quantities. The amount of fly ash production, in the world, is about 500 million tons every year [1]. The largest coal producing countries are not confined to one geographical region and the top five hard coal producers/consumers are China, India, USA, Indonesia, and Australia [2,3]. The World coal consumption fell by 4.2%, its fourth decline in six years [3].

Currently, there is interest in utilizing bottom and fly ash particles in various industrial processes, for example, in road and pavement construction [4,5], as an admixture to metal or plastics, e.g., metal syntactic foam [6], metal-matrix composites [7,8], or as materials for biotechnology [9]. These applications stimulate the studies on the properties of fly ash particles. In particular, cenospheres, which are hollow spherical-shaped, aluminosilicate particles of micro- and nanometer diameters with high tensile strength properties are modern thermal insulation materials of light weight. Due to their low weight, high hardness, and compressive strength, alloys with fly ash particles are widely used in automotive or space structures [10].

Other types of fly ash particles are those with magnetic properties, i.e., high magnetic susceptibility, which can be used in specific applications, for example, as catalysts with specific catalytic and sorption properties, for wastewater treatment [11] or Hg0 removal from flue gases [12]. The advantage of using magnetic catalysts is that the magnetic particles can be recovered from the waste of the process by using a magnetic field, regenerated and reused. One of the cheaper sources of such magnetic particles is coal fly ash. Separation of magnetic particles (also called magnetospheres or ferrospheres in different publications), with specific properties from the fly ash may contribute to the determination of the method of their use in order to conduct a circular economy.

Magnetic particles of fly ash are small, usually hollow spheroids roughly 1–100 µm in diameter, which constitute about 1–2 wt.% of the coal combustion products. The magnetic crystallites on the particles are blocky, skeletal, dendritic, and plate-like structures, with different sizes and shapes, and are randomly deployed on the surface [13].

Experimental results have shown that the yield of ash magnetic particles varies depending on combusted coal and the stage of power plant, and can be in the range of 1–14% [1,12]. Magnetic particles that originated from fly ash can be classified into four groups, depending on the iron content, which is shown in Table 1 [14].

Magnetospheres are considered to be nanocomposites of “core-shell” structure with glass matrix, where the core is mainly composed of silica and the shell is rich in spinel, hematite, and quartz crystals [1]. The main iron-containing phases at the surface are usually identified as Fe_3_O_4_, α-Fe_2_O_3_, γ-Fe_2_O_3_, Fe^2+^-silicate, Fe^3+^-silicate, and FeS; their contents vary significantly in different magnetospheres [14,15].

In this paper, the properties of magnetic particles derived from fly ash particles were analyzed. Magnetic particles used in this research were formed during combustion of pulverized anthracite coals from the Upper Silesian region in Poland in a pulverized coal boiler at the “Opole” power station (Southwest Poland).

Fly ash particles were analyzed by various methods for the determination of their morphology and morphology of their surface, elemental composition, phase content, and particle size distribution. The mineral composition, chemical composition, and microstructure of magnetic particles were studied by X-ray diffraction (XRD), energy dispersion spectroscopy, and scanning electron microscopy, respectively.

## 2. Research Methodology

### 2.1. Sample Preparation

**Figure 1:**
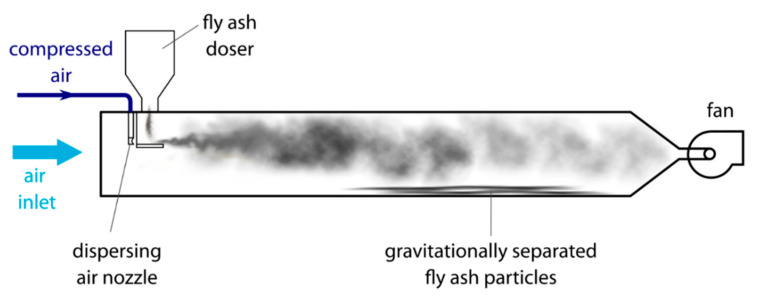
Scheme of experimental channel used for particle separation.

The fly ash used in this research was collected from electrostatic precipitator (ESP) hoppers at a coal-fired power plant in Poland. Particles from the 1st field were only analyzed for magnetic properties. The separation of magnetic particles from the bulk of fly ash was a two-step process. In the first step, the fly ash was dispersed into a horizontal channel of 160 × 160 mm cross-section and length of 1.8 m made of acrylic glass, via pouring the fly ash from a LAMBDA dosing system (LAMBDA Laboratory Instruments, Baar, Switzerland, 1 L vessel volume) onto a tray mounted in the middle of the cross-section of the channel, and dispersed by compressed air flowing from a flat nozzle onto this tray under a pressure of 3 bar. The scheme of the channel is shown in Figure 1. Lighter particles were conveyed by the flowing air to the outlet, whereas heavier particles fell onto the bottom of the channel. In the next step, magnetic particles were recovered by dry magnetic separation from the fly ashes deposited on the bottom of the channel using a handheld neodymium magnet. For further cleaning, the separated magnetic fractions with a nonmagnetic fly ash particle mixture were drawn onto a paper sheet and the handheld magnetic separation was repeated several times.

### 2.2. Particle Morphology and Elemental Composition

The morphology and elemental composition of magnetic particles were investigated under a scanning electron microscope (SEM) Zeiss EVO 40 (Carl Zeiss, Oberkochen, Germany) equipped with an energy dispersive spectroscopy (EDS) detector SDD X-flash 5010, 10 mm^2^, 125 eV, Bruker Quantax 400 (Bruker GmbH, Berlin, Germany). The elemental composition of bulk fly ash was obtained via scanning three different areas of about 10 × 10 µm, or circles of different sizes marked on micrographs. Because of the limited sensitivity of this detector, only elements of atomic abundance higher than 0.1 at.% were identified. The EDS detector was capable of detecting elements from those with atomic numbers equal or higher than 4 (beryllium) to americium (atomic number 95). The intensity of each peak of the EDS spectrum is a quantitative measure of the element concentration. The particles to be investigated under the scanning electron microscope were poured onto a microscopic table coated with conductive carbon tape. To form a conductive coating on the particles’ surfaces, a ~20 nm thick gold and platinum layer was spattered onto the surface of the powder samples using a sputter coater Emitech K550X (Quorum Technologies Ltd., Ashford, UK).

### 2.3. Particle Size Distribution

The size distribution of magnetic particles was determined from SEM micrographs taken with a magnification of ×5000, via measuring the diameter of more than 2500 particles, using the NIS Elements Imaging Software (Nikon, Tokyo, Japan, version 3.21.00). The obtained sizes were classified into 99 classes in the range from 100 nm to 10 µm with D_i_ = 0.1 µm interval. The micrograph samples were taken from various places on the fly ash specimens, and at least ten micrographs for each type of fly ash were analyzed.

### 2.4. Mineralogical Composition

The mineralogical composition of the magnetic particles was detected by an X-ray diffraction system, diffractometer Xpert PRO-MPD Philips (2003, Almelo, The Netherlands) with copper K_α_ radiation (*λ* = 1.5404 Å), in the scattering angle range of 2*θ* = 20–80°. The constituent crystal phases of the particles were identified busing the HighScore Plus software (Version 3.0e (2012)) operating with the diffractometer.

## 3. Results

### 3.1. Particle Size Distribution

**Figure 2:**
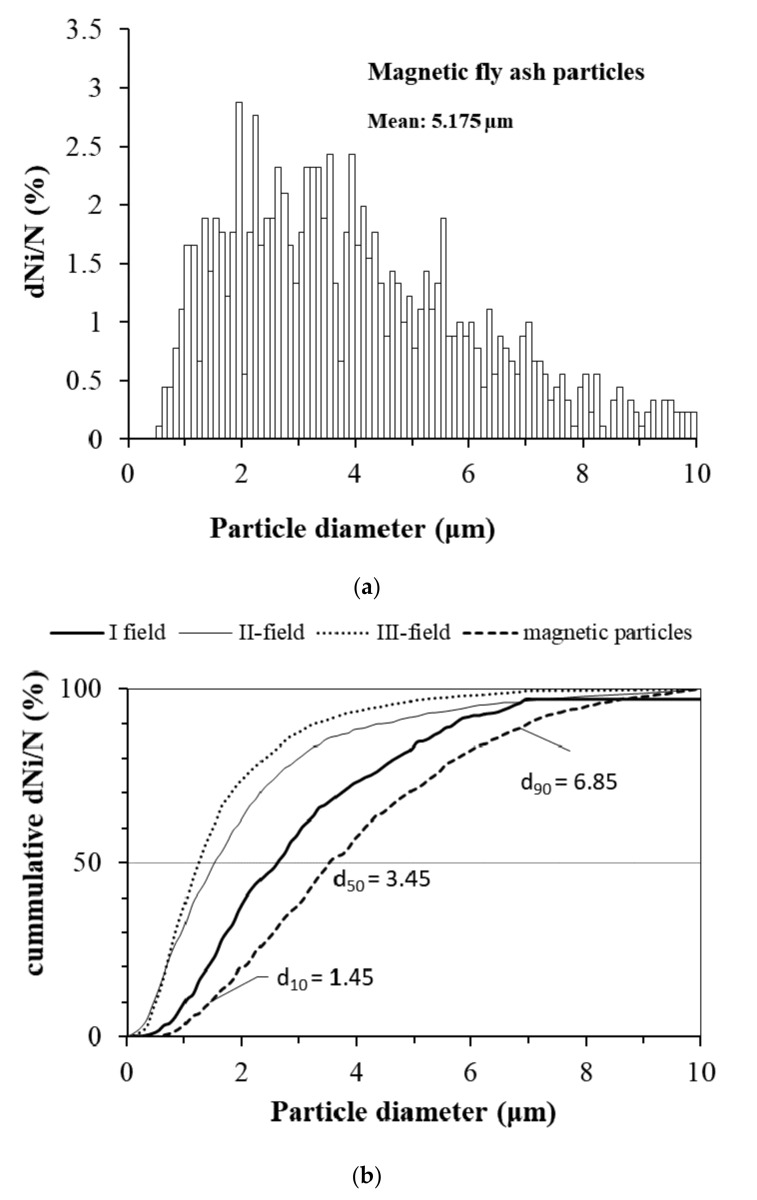
(**a**) Size distribution of the magnetic particles; (**b**) comparison of cumulative number size distributions of magnetic particles and fly ash particles from three fields of the electrostatic precipitator.

The size distribution of the magnetic particles separated from fly ash of ESP is shown in Figure 2a. Figure 2b compares the cumulative number size distribution of magnetic particles with spherical fly ash particles from three fields of ESP. The average size of the magnetic particles is about 5 µm, and it is larger than the average fly ash particle size (1.25, 1.5, and 2.65 µm for I, II, and III ESP field), but single magnetic particles of size up to 100 µm can also be found. The median diameter of the magnetic particles is d_50_ = 3.45 µm.

### 3.2. Particle Morphology

**Figure 3:**
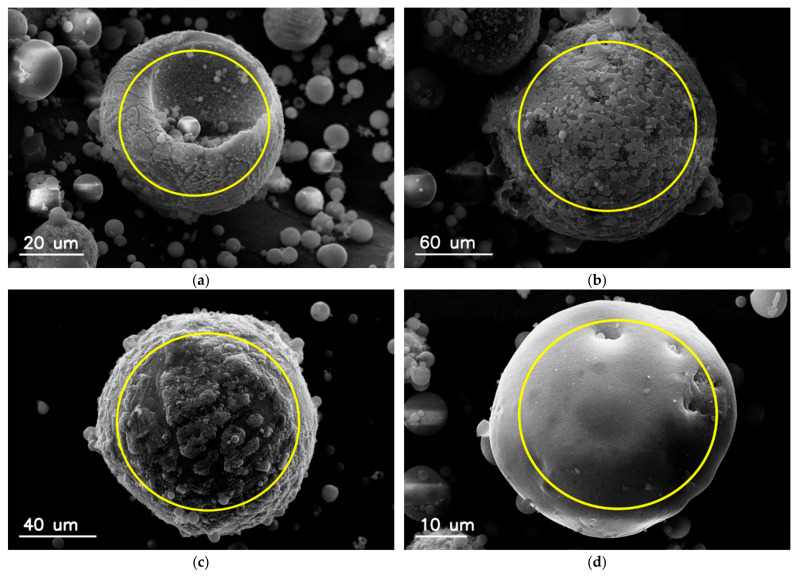
SEM images of individual magnetic particles recovered from fly ash and the corresponding iron content: (**a**) 51.15; (**b**) 29.08; (**c**) 16.30; (**d**) 2.02 (in wt.%). The scanning area of electron beam for EDS measurements is marked with a circle in each image.

Various micrographs of magnetic particles taken under the scanning electron microscope are shown in Figure 3. It can be noticed that, unlike aluminosilicate particles, magnetic particles are not ideal spheres. The surface of the magnetic particles is also not smooth. The morphology of the aluminosilicate particles that comprise the majority of fly ash with small iron content (Figure 3d) differs significantly from other fly ash magnetic particles (Figure 3a–c).

**Figure 4:**
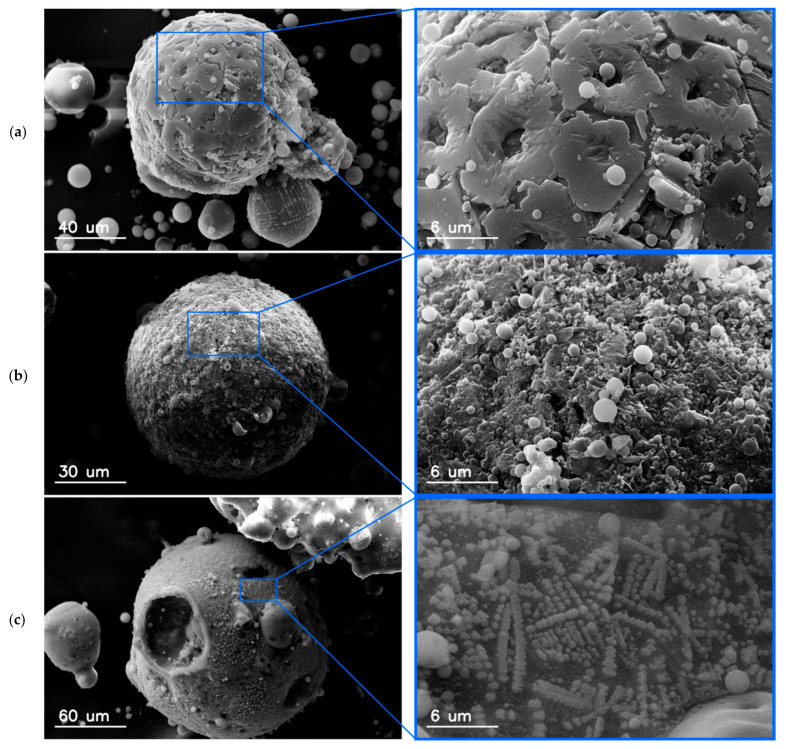
SEM micrographs of surface morphology of magnetic particles: (**a**) 45.95; (**b**) 42.38; (**c**) 49.6 (in wt.%).

Figure 4 shows the surface morphology of the magnetic particles. The surface is not smooth in the close-up view, but corrugated and/or covered with various types of smaller particles, flakes, or polyhedral structures, which are partially merged with the mother particle, or with the submicron spherical aluminosilicate particles. Contrary to aluminosilicate particles, which are in mostly almost ideal spheres with glassy surface, the magnetic particles often have spinel-like or crystal-like structures adhered to the surface.

There is a relationship between the shape and surface microstructure of particles, and the iron content. The content of iron compounds in particles with a smooth surface is significantly lower than in irregular particles. This effect suggests different mechanisms of the formation of magnetic particles compared to other aluminosilicate particles (Figure 4).

### 3.3. Magnetic Particles’ Elemental Compositions

The elemental compositions of the magnetic and nonmagnetic particles are compared in this section. There is a lack of criterion by which a fly ash particle can be classified as magnetic or nonmagnetic. Kukier et al. [16] classified particles with the Fe_2_O_3_ percentage less than 5.46 wt.% as weakly magnetic and nonmagnetic. The percentage of iron in nonmagnetic fractions ranged from 1.5 to 4.43% in their investigations. Fomenko et al. [17,18] investigated the compositions and morphology of magnetic and nonmagnetic cenospheres derived from fly ash, and classified those which contained 2.6–3.5 wt.% of Fe_2_O_3_ as nonmagnetic. For the purpose of this research, it is arbitrarily assumed that nonmagnetic particles contain less than 5 wt.% of Fe_2_O_3_, which is equivalent to 3.5 wt.% of Fe.

**Figure 5:**
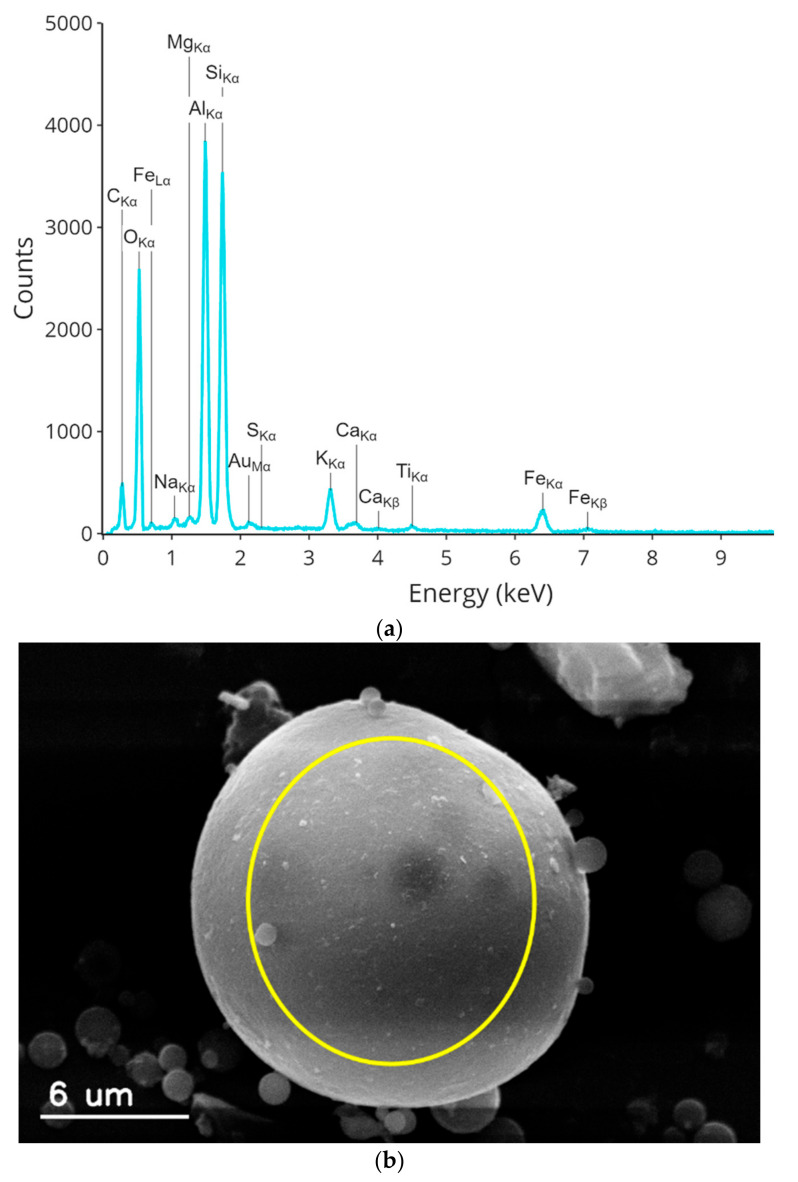
(**a**) EDS spectrum of a nonmagnetic particle; (**b**) SEM microphotograph with circle showing the area the spectrum acquisition.

The EDS spectrum of the elemental composition of nonmagnetic particles is shown in Figure 5. The iron peak occurs at 6.38 eV, and the Fe content was about 3%. The elemental composition of a spherical nonmagnetic particle from Figure 5b (in wt.%) is summarized in Table 2.

The elemental composition of the nonmagnetic fly ash particles obtained by the EDS analysis indicates that the main components are SiO_2_ (21.5 wt.%) and Al_2_O_3_ (24.9 wt.%). Iron inclusions are dispersed on the surface of aluminosilicate particles, while other compounds (calcium, sulfur, phosphorus, titanium, and manganese) are the doping compounds associated with the remaining particles.

**Figure 6:**
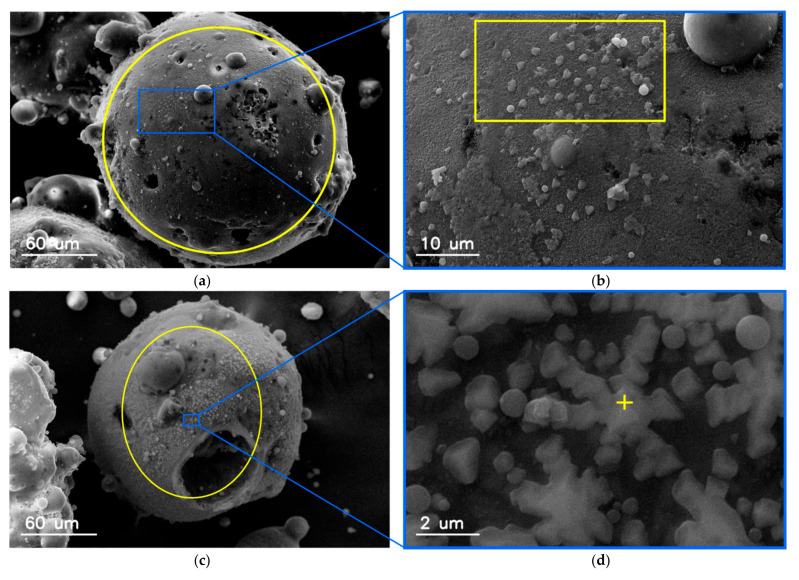

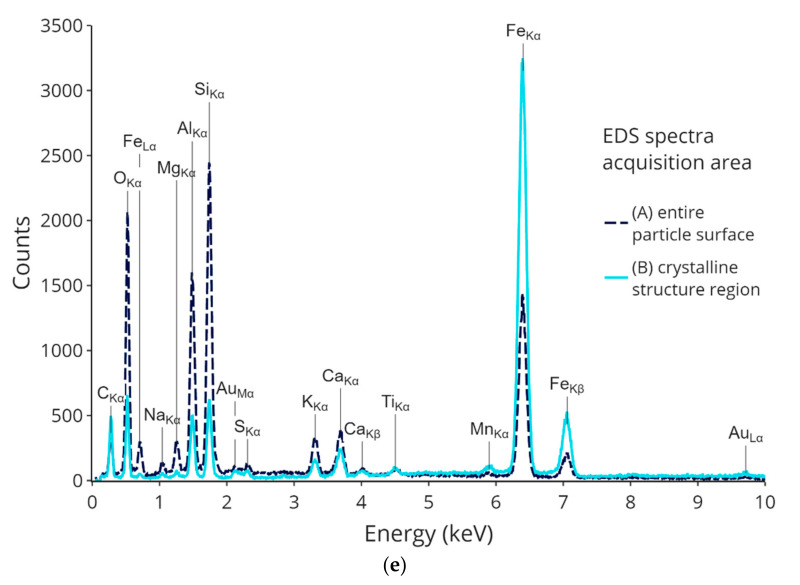
SEM micrographs of magnetic fly ash particles and its EDS spectra: (**a**,**c**) General views of the selected particles with marked area of EDS spectrum acquisition; (**b**,**d**) detailed views of crystalline structures visible on the (**a**,**c**) particles. respectively; (**e**) EDS spectra of particles shown in (**a**,**b**).

Examples of the SEM micrographs of magnetic fly ash particles and the EDS spectra are shown in Figure 6. The area from which the EDS spectrum was taken is marked with a circle. The magnetic crystallites on fly ash particles are not homogeneously deposited, but the magnetic crystalline inclusions are randomly dispersed on the surfaces of mother aluminosilicate particles. The magnetic crystals agglomerated on the particle surface are of different morphology. Figure 6e presents the EDS spectra taken from different fragments of the magnetic particles.

The regular nano-sized grains on the surface of particle can be dispersed separately (Figure 6b) or coagulated, forming permanently different patterns, for example, bound chains (Figure 6d).

The concentrations of elements forming a magnetic particle, such as C, O, Si, Na, Mg, Al, Si, P, S, Ca, Ti, Mn, Fe, and Cu determined by the EDS method are presented in Table 3. Magnetic particles are also rich in alkaline (calcium, magnesium, and potassium) compounds.

The SEM-EDX study of individual magnetic particles showed that the major components are silicon, aluminum, and iron; the sum of Si and Al varied from 15 to 40 wt.% with the average of 20 wt.%, in the majority of particles. The other elements in magnetic particles are a mixture of aluminosilicates with moderate amounts of Ca, Mg, Na, and Mg, and trace elements such as Mn and Ti. The EDX analysis indicated that all of these compounds appear as oxides. The concentration of trace elements (Ni, Ba, Cu, and Mo) in all samples is relatively low (Table 3).

**Figure 7:**
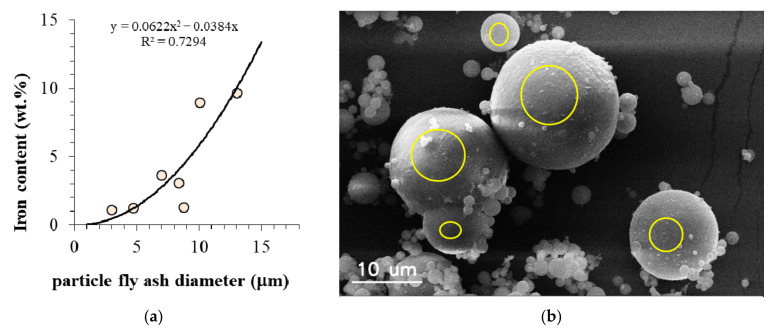
(**a**) The relation between the magnetic particle size and iron content in it; (**b**) SEM microphotographs with marked area of EDS spectra acquisition for various particles.

Figure 7 shows the correlation between the size of a magnetic particle and the iron content in wt.%. This plot shows the trend of increasing iron content in magnetic particles with increasing diameters of the particles.

The results presented in Figure 7 for magnetic particles of various sizes show that the iron content relation can be described by a second-degree regression curve, with correlation coefficient R^2^ = 0.73, which suggests that the iron content is proportional to the surface area of the particle, probably, as a result of iron vapors condensation on the entire surface of aluminosilicate particle during gas cooling.

**Figure 8:**
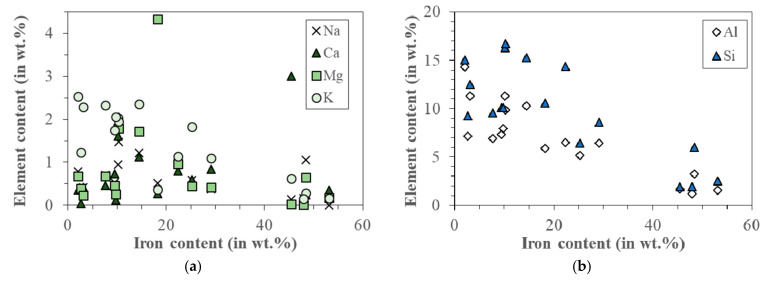
(**a**) Correlations between potassium, sodium, calcium, magnesium, content (in wt.%) and the iron content in magnetic particles; (**b**) dependence of silicon and aluminum content in individual magnetic particles as a function of iron.

The correlations between the concentration of different elements in magnetic fly ash particle (potassium, sodium, calcium, magnesium, silicon, and aluminum content in wt.%) and the iron content are shown in Figure 8 and summarized in Table S1. With increasing iron content, the concentration of all elements, except calcium, decreases, for example, with increasing iron concentration in the range from 5 to 60 wt.%, In highly magnetic particles (aluminosilicate-bearing ferrooxides, following the Zhao [14] classification), the concentrations of aluminum and silicon each drop below 5 wt.% (Figure 8b).

### 3.4. Magnetic Particles Mineralogy

**Figure 9:**
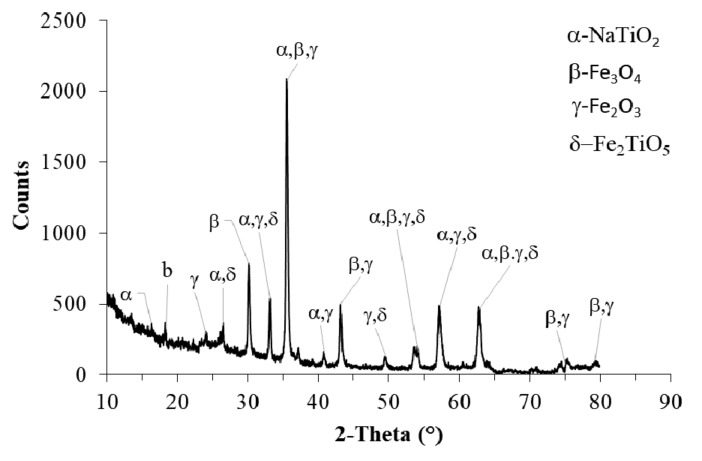
X-ray diffractogram of fly ash from ESP after combustion of coal in a pulverized fuel boiler.

The X-ray diffractogram of fly ash particles is shown in Figure 9. The XRD patterns provide information on the particle size and defects, while the peak relative intensities provide insight into the atomic distribution in the unit cell. For the correct interpretation of powder diffractograms, a good peak-to-background ratio is an important issue. The analysis of XRD patterns and diffraction peaks characterizes the crystalline phase of the materials.

The XRD results show that the dominant phase is magnetite Fe_3_O_4_ and maghemite γ-Fe_2_O_3_ (with a considerable amount of Fe-spinel enriched by Na and Ti elements, cf. Figure 9).

## 4. Discussion

The process of magnetic particle formation during the combustion of coal is still an unsolved problem. The presence of magnetic particles on the surfaces of aluminosilicate particles (Figure 4 and Figure 6) suggests that iron compounds are deposited on them during gas cooling. However, Xing and Rosner [19] suggested that the formation of nanoparticles from the gas phase consisted of several, sometimes purely sequential processes. First, chemical reactions take place in the oxidizing atmosphere of exhaust gases and iron vapors are oxidized to FeO or Fe_2_O_3_, then, nucleation of supersaturated vapors form embryonic particles, the primary particles of nanometer size, which grow by vapors condensation or heterogeneous chemical reactions. During this process, other oxides produced in the flames (for example of those shown in Figure 8, mainly SiO_2_, Al_2_O_3_, or TiO_2_) adhere to these nuclei. The magnetic particles are the result of transformation of iron containing minerals with aluminosilicates, which have been connected by alkaline compounds (calcium, magnesium, or potassium).

The iron content in the bulk magnetic particles can be up to 60 wt.% [15,20], which is confirmed by the present results. The mineralogical forms of iron crystals embodied in the magnetic fly ash particles is also an open issue, although the concentration of iron compounds in magnetic particles has been investigated by several authors. The dominant form detected in this study was hematite Fe^+3^ (Fe_2_O_3_), but other magnetic crystallites, such as Fe_3_O_4_, α-Fe_2_O_3_, γ-Fe_2_O_3_, Fe^2+^-silicate, Fe^3+^-silicate, and FeSi were also detected. The content of each components varied significantly in different magnetic particles [14].

## 5. Conclusions

In this study, the composition and properties of individual globular magnetic particles collected from an electrostatic precipitator and separated from the bulk fly ash using a handheld magnet were investigated. The size distribution of magnetic particles with different iron contents ranged from about 0.2 µm to 100 µm, and their mean size was 5.176 µm. The magnetic particles were characterized by a high iron content of percentage ranging from 5 to 52 wt.%.

It was shown that the iron content of each particle was proportional to its surface, i.e., it was a square function of the particle radius, with high probability R^2^ = 0.73. This suggests that iron oxides are mainly deposited as an effect of vapor condensation of iron with subsequent oxidation or as adhesion of oxidized iron crystallites on aluminosilicate particles. It was found that hematite Fe^+3^ (Fe_2_O_3_) was the dominant form of crystallites detected on the aluminosilicate fly ash particles. On the surface of the magnetic particles, one could also notice many agglomerates, spherical nanoparticles, fine crystalline fibers, and crystals.

## Figures and Tables

**Table 1:** Types of magnetic particles in fly ash formed during coal combustion [14].

Type of Magnetic Particles	Iron Content (wt.%)
ferrooxides	Fe ≥ 75%
Aluminosilicate-bearing ferrooxides	75% ≥ Fe ≥ 50%
Ferriferous aluminosilicates	50% ≥ Fe ≥ 25%
Ferro-aluminosilicates	25% ≥ Fe

**Table 2:** Average elemental composition of the spherical nonmagnetic particle from Figure 5 (in wt.%).

C	O	Na	P	Al.	Si	Cu	S	Fe	Mg	Mn	Ti	Ca	K
19.35	49.84	0.41	0.00	11.29	12.48	0.13	0.03	3.06	0.23	0.01	0.37	0.41	2.28

**Table 3:** The elemental compositions (in wt.%) of the magnetic particles shown in Figure 6.

EDS Spectrum Area	C	O	Na	P	Al.	Si	Cu	S	Fe	Mg	Mn	Ti	Ca	K
A	15.45	45.79	0.49	0.01	7.15	10.69	0.11	0.17	14.29	1.54	0.12	0.32	2.22	1.65
B	24.93	20.20	1.63	0.00	7.45	8.28	0.21	0.53	31.56	1.10	0.35	0.34	2.00	1.40
C	8.16	44.25	1.21	0.01	10.30	15.28	0.16	0.05	14.43	1.72	0.07	0.47	1.12	2.35
D	2.84	39.83	1.12	0.08	6.83	9.08	0.21	0.19	34.53	2.43	0.15	0.28	1.10	1.32

## Data Availability

No supporting data.

## Supplementary Materials

The following supporting information can be downloaded at: https://www.mdpi.com/article/10.3390/ma15020528/s1, Table S1: Content (in wt.%) individual compounds of magnetospheres presented in Figure 8.
