# Supplementary material for: Morphology and Chemical Composition of Magnetic Particles Separated from Coal Fly Ash
Source: Materials (Basel). 2022 Jan 11;15(2):528. doi: 10.3390/ma15020528 (PMC8779241; doi:10.3390/ma15020528)
Supplement: Supplementary file 1 [file materials-15-00528-s001.zip › materials-1480843-supplementary.pdf]

materials

Supplementary materials

Morphology and chemical composition of magnetic particles separated from coal fly ash

Tadeusz Czech

Institute of Fluid Flow Machinery, Polish Academy of Sciences, Fiszera 14. 80-231 Gdańsk, Poland
* Correspondence: author: plasdyn@imp.gda.pl

Table S1. Content (in wt.%) individual compounds of magnetospheres presented in Figure 8.

Particle Number	1	2	3	4	5	6	7	8	9	10	11	12	13	14	15	16	17
Carbon	29.61	5.60	13.55	15.00	8.69	9.55	23.73	8.62	6.13	10.11	8.16	18.95	21.20	7.37	12.84	7.23	19.35
Oxygen	48.30	33.63	49.76	32.96	33.14	42.80	46.12	46.41	48.07	48.27	44.25	47.02	47.69	44.78	46.42	38.27	49.84
Sodium	0.41	1.05	0.78	0.16	0.00	0.37	0.70	0.94	1.47	0.58	1.21	0.52	0.42	0.99	0.51	0.13	0.41
Magnesium	0.39	0.65	0.67	0.02	0.18	0.42	0.68	1.79	1.78	0.45	1.72	0.45	0.26	0.96	4.33	0.03	0.23
Aluminium	7.15	3.24	14.29	1.18	1.54	6.42	6.92	11.29	9.85	5.15	10.30	7.31	7.96	6.50	5.88	1.69	11.29
Silicon	9.28	6.01	15.01	1.92	2.53	8.59	9.54	16.30	16.74	6.43	15.28	10.12	10.13	14.35	10.56	1.92	12.48
Phosphorus	0.03	0.03	0.17	0.19	0.01	0.15	0.10	0.05	0.01	0.09	0.01	0.01	0.00	0.04	0.00	0.72	0.00
Sulfur	0.06	0.00	0.05	0.06	0.03	0.00	0.82	0.02	0.75	0.64	0.05	0.00	0.00	0.04	0.00	0.43	0.03
Chlorine	0.00	0.00	0.00	0.00	0.02	0.00	0.01	0.00	0.00	0.00	0.00	0.00	0.00	0.00	0.00	0.09	0.00
Potassium	1.23	0.28	2.53	0.15	0.16	1.09	2.33	2.03	1.95	1.83	2.35	1.74	2.05	1.14	0.37	0.62	2.28
Calcium	0.04	0.24	0.35	0.15	0.35	0.83	0.46	1.61	2.06	0.59	1.12	0.72	0.12	0.79	0.26	3.01	0.41
Barium	0.04	0.10	0.16	0.00	0.02	0.07	0.10	0.05	0.16	0.03	0.42	0.54	0.00	0.01	0.00	0.04	0.10
Titanium	0.54	0.36	0.46	0.04	0.10	0.35	0.75	0.53	0.50	0.38	0.47	2.97	0.27	0.43	0.25	0.14	0.37
Manganese	0.06	0.00	0.02	0.00	0.00	0.00	0.02	0.00	0.14	0.00	0.07	0.00	0.00	0.11	0.17	0.18	0.01
Iron	2.64	48.41	2.01	47.89	53.08	29.12	7.62	10.19	10.26	25.27	14.43	9.48	9.75	22.35	18.25	45.39	3.06
Cooper	0.22	0.40	0.18	0.27	0.15	0.23	0.10	0.17	0.13	0.18	0.16	0.16	0.15	0.14	0.14	0.10	0.13
